# Flavor improving effects of cysteine in xylose–glycine–fish waste protein hydrolysates (FPHs) Maillard reaction system

**DOI:** 10.1186/s40643-023-00714-8

**Published:** 2023-12-21

**Authors:** Yicheng Ding, Chen Yan, Wangli Dai, Yanbo Wang, Shulai Liu, Renchao Zheng, Xuxia Zhou

**Affiliations:** 1 Key Laboratory of Marine Fishery Resources Exploitment & Utilization of Zhejiang Province, College of Food Science and Technology, Zhejiang University of Technology, Hangzhou, 310014 People’s Republic of China; 2 Key Laboratory of Bioorganic Synthesis of Zhejiang Province, College of Biotechnology and Bioengineering, Zhejiang University of Technology, Hangzhou, 310014 People’s Republic of China; 3 Beijing Advanced Innovation Center for Food Nutrition and Human Health, Beijing, Key Laboratory of Flavor Chemistry, Beijing Technology and Business University, Beijing, 100048 People’s Republic of China

**Keywords:** Fish protein hydrolysates (FPHs), Maillard reaction, Cysteine, Volatile compounds, Mantel test

## Abstract

A promising way to utilize fish by-products is to develop hydrolysis of fish proteins with enzymes. The obtained fish protein hydrolysates (FPHs) are rich in peptides and amino acids, but bitterness and aroma defects impede further utilization of FPHs. The present study adopted Maillard reaction to improve FPHs’ flavor and illustrated the role of cysteine in this system. We investigated the impact of cysteine (0, 0.25%, 0.5%, 0.75%, and 1%) on the browning intensity, free amino acids (FAAs), molecular weight distribution, structure of MRPs, volatile compounds changes and organoleptic characteristics of xylose–glycine–FPHs Maillard reaction systems. Results showed that the addition of cysteine lowered the browning degree of Maillard reaction products (MRPs) by inhibiting the cross-linking of small peptides and reducing the production of melanin. GC–MS and GC–IMS analysis indicated that cysteine inhibited the formation of furans and nitrogen-containing compounds and facilitated the formation of sulfur-containing compounds contributing to the meaty flavor. Sensory analysis revealed that 0.25–0.75% range of cysteine increased the meaty, caramel, umami, mouthfulness and salty notes, and caused a decrease in bitter taste of the MRPs as confirmed by GC–MS. A highly significant correlation between the organoleptic characteristics and physicochemical indicators of MRPs was found by Mantel test. These results elucidated the influence of cysteine on the formation of Maillard reaction products and will help improve the flavor profile of meat flavorings.

**Graphical Abstract:**

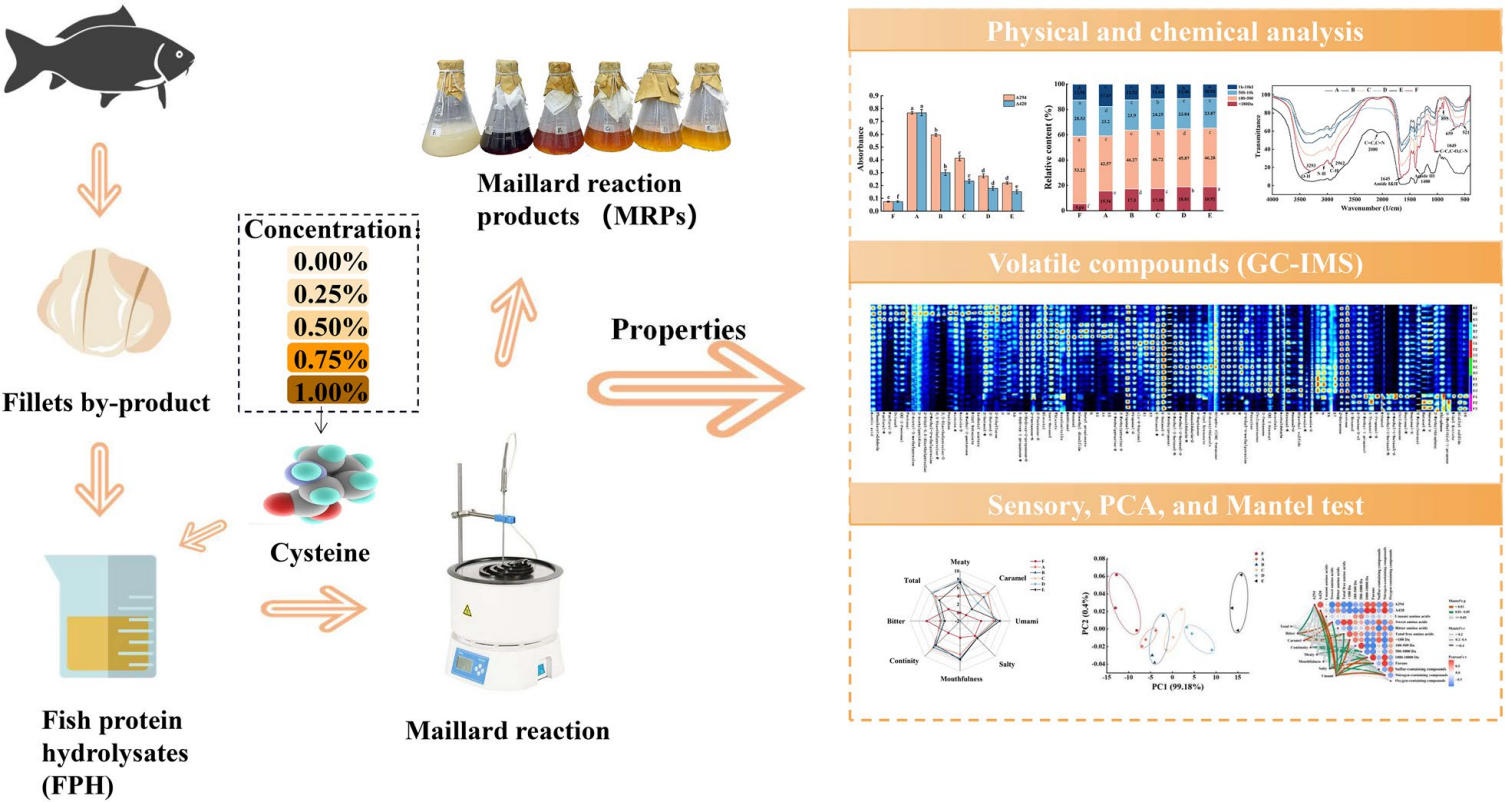

## Introduction

Seafood is becoming more popular with consumers for healthy lifestyles, due to its high-quality protein content, rich unsaturated fatty acids, and important trace elements. However, the fishery industry produces large quantities of fish by-products, such as heads, fins, viscera, and sometimes muscle. The by-products derived from fish typically constitute a substantial portion, ranging from 50 to 90% of the total mass, depending on the species and intended use. Unfortunately, most of these by-products are discarded, with only a fraction being utilized for low-value purposes such as feed and fertilizer (Zhou et al. 2023). To utilize these protein-rich fish by-products, different enzymes, such as alkaline protease and flavor enzymes, are employed to developed hydrolysis of fish proteins (Gao et al. 2021). The fish protein hydrolysates (FPHs) are a composition of amino acids and peptides with molecular weights less than 30 kDa (Je et al. 2005). In comparison to the original proteins, FPHs are marketed as a dietary supplement and has been purported to have various health-enhancing benefits. Numerous functional and nutritional properties have been observed in FPHs, including antioxidant, anti-microbial, anti-hypertensive, anti-diabetic, anti-inflammatory, anti-cancer, and anti-obesity properties (Alahmad et al. 2022). In addition to its other beneficial qualities, FPHs exhibit desirable physical and chemical properties, including superior solubility, the ability to foam and emulsify, and an impressive water- and oil-binding capacity (Siddik et al. 2021).

However, bitterness and aroma defects are common obstructions for FPHs to be widely utilized as a nutritive food ingredient. The bitterness of FPHs is associated with degree of hydrolysis, hydrophobicity, proline residues, and molecular weight, and peptides containing bulky hydrophobic groups towards the C-terminal have been identified as the primary contributors to bitterness (Idowu and Benjakul 2019). The aroma defects of FPHs came from volatile compounds associated with aldehydes and lipid oxidation (Liu et al. 2022). To decrease bitterness and promote the aroma of FPHs, Maillard reaction appears to be a promising method. The Maillard reaction, also known as non-enzymatic browning, involves reactions between the carbonyl groups in reducing sugars and the amino groups of proteins, peptides, and amino acids (Gao et al. 2020). The Maillard reaction plays an important role in the generation of a variety of volatile compounds, non-enzymatic intermediates, and high molecular weight melanoidins, which contribute dramatically to the aroma, color, taste, and antioxidant properties of foods (Sun et al. 2022). Studies have been conducted to prepare flavors with the FPHs by Maillard reaction. However, most studies mainly focused on the optimization of hydrolysis process and Maillard reaction conditions, few researchers have been made to elucidate the formation of aroma and taste components from FPHs during the Maillard reaction (Gao et al. 2020; Yu et al. 2022).

The Maillard reaction products (MRPs) are responsible for the taste and aroma of the reaction, as their compositions are influenced by substrates in the reaction system. Tastes related MRPs are mainly Maillard-reacted peptides, which are reported to show salt taste-enhancing properties (Yu et al. 2022). Similarly, aroma related MRPs mainly depend on the types of peptides, amino acids and reducing sugars in the system, and reaction conditions such as temperature, time, and pH (Fadel et al. 2023). Studies have shown that cysteine can significantly enhance the formation of MRPs with meat-like flavor (Fadel et al. 2023; Gao et al. 2020). Many studies presented cysteine as a flavor precursor due to its contribution to the production of meaty aroma compounds, such as pyrazines and 2-methyl-3-furanthiol, through pyrolysis or Strecker degradation with dicarbonyl compounds (Gao et al. 2020; Yu and Zhang 2010).

Many simple model Maillard reactions of a single reducing sugar and a single amino acid have been also investigated to evaluate the formation of meat flavors (Gao et al. 2020; Zhang et al. 2022; Zheng et al. 2023). However, few studies have reported the effects of cysteine on MRPs in the glycine–xylose reaction using FPHs as substrates. Therefore, in this study, with the FPHs as the source of basic amino acids and peptides, reaction system composed of glycine–xylose with different amounts of cysteine or without cysteine were designed and performed, and the effect of cysteine on browning intensity, free amino acids (FAAs), molecular weight distribution, structure of MRPs, volatile compounds changes and organoleptic characteristics of the MRPs were investigated. Furthermore, correlations between browning intensity, FAAs, molecular weight distribution, volatile compounds, and organoleptic properties of MRPs were explored by mantel test.

## Materials and methods

### Materials and chemicals

Snake-headed fish fillet processing waste used for the preparation of protein hydrolysate was obtained from Qiandao Lake Group Co., Ltd. (Hangzhou, China). Alkaline protease (30,000 U/g), flavor protease (30,000 U/g), L-cysteine, L-glycine, and D-xylose were obtained from Henan Wanbang Chemical Technology Co., Ltd. (Henan, China). Other chemicals were all analytical reagents and purchased from China National Pharmaceutical Group Co. (Sinopharm, Shanghai, China).

### Preparation of snake-headed FPHs from fish waste

Snake-headed fish waste was minced and then 100 g of the mince was dispersed in 300 mL of distilled water at a water/substrate ratio of 3:1. The mixture was then blended and homogenized to facilitate the hydrolysis reaction. The pH of the mixture was adjusted to 7 using 2 M NaOH, then alkaline protease and flavor enzyme were added in a ratio of 2:1, with a total amount of 3500 U/g. The hydrolysis reaction was carried out for 5.5 h in a water bath at 55 °C with continuous shaking (100 rpm). After hydrolysis, the enzymes were inactivated by heating the suspension at 100 °C for 15 min in the water bath. The sample was immediately cooled to room temperature in ice water. The enzymatic hydrolysate was further subjected to centrifugation at 8,000 g for 15 min at 4 °C. The supernatants were collected and stored at −80 °C until further use.

### Maillard reactions

Maillard reaction was carried out according to the previous method (Zhang et al. 2018) with some modifications. A 10 mL solution reaction system consisting of hydrolysate, D-xylose (0.25%), and L-cysteine (0–1%) were prepared in a beaker. The solution was adjusted to pH 7.0 with 1 mol/L NaOH. The solution was transferred into sealed glass tubes and reacted in oil bath at 110 °C for 60 min. MRPs with 0%, 0.25%, 0.5%, 0.75% and 1% of L-cysteine were named groups A, B, C, D, and E, respectively. Finally, the MRPs were placed in ice water and portions of samples were freeze-dried while the others were stored at − 20 °C for further analysis. The heated product without added L-cysteine (group A) was used as negative control and the unheated product was used as blank control (group F). Samples were prepared in three replicates for further analysis.

### Measurement of intermediate products and browning intensity

To evaluate the formation of intermediate products of non-enzymatic browning and the brown polymers formed in more advanced stages of the MRPs, the intermediate products and browning intensity of all the MRPs solutions were determined by a UV–Vis spectrophotometer (T10CS; Puxi Instrument Co., Ltd. China) (Wang et al. 2019), using a 50-fold dilution with ultrapure water at 294 nm and 20-fold dilution at 420 nm, with ultrapure water as a blank reference.

### Determination of free amino acids

The content of FAAs was determined and analyzed by automatic amino acid analyzer (Biochrom 30 + ; Tokyo, Japan). Samples were treated according to previous methods (Liu et al. 2019). Proteins or peptides in MRPs were precipitated by adding an equivalent volume of 5% (m/v) sulphosalicylic acid at 4 °C for 2 h, and then centrifuged at 8000 r/min at 4 °C for 10 min. The pH of the supernatant was adjusted to 2.0 with 6 M NaOH and filtered with a 0.45 μm microfiltration membrane before amino acid analysis. A calibration curve was obtained using the standard mixture of amino acids from Sigma-Aldrich (St. Louis, MO), and the content of each amino acid was calculated based on its retention time and peak area.

### Estimation of molecular weight (MW) distribution

The molecular weight (MW) distribution profiles of the MRPs were estimated by high-performance gel-filtration chromatography (Siewe et al. 2020). Waters 1525 liquid chromatography system (Waters Co., Milford, MA, USA) equipped with a 2487 UV detector and an Empower workstation was used for this experiment. The column used was TSK gel 2000 SWXL 300 mm × 7.8 mm (Tosoh Co., Tokyo, Japan), and the mobile phase consisted of acetonitrile/water/trifluoroacetic acid (45/55/0.1, v/v/v) was delivered at a flow rate of 0.5 mL/min. Each sample was diluted to a concentration of 10 mg/mL with mobile phase and filtered through a 0.45-μm syringe filter before loading. The column temperature was 30 °C, and 10 mg of each sample was injected into the HPLC system for the analysis. An MW calibration curve was obtained from the following standards from Sigma: cytochrome C (12,400 Da), bacitracin (1450 Da), tetrapeptide GGYR (451 Da), and tripeptide GGG (189 Da). The results were obtained using a UV detector (220 nm), and the data analysis was performed using gel permeation chromatography (GPC) software.

### Fourier transform infrared spectroscopy (FTIR) analysis

The infrared analysis was performed using FTIR spectroscopy (Thermo Scientific Nicolet iS20). The lyophilized sample was uniformly mixed with dry KBr at a weight ratio of 1:100. All the spectra were an average of 32 scans from 4000 to 400 cm^−1^ at a resolution of 4 cm^−1^ (Liu et al. 2019). The background noise was corrected with the data of pure KBr. The raw FTIR data were processed with Omnic spectrum software (Version 9.2.106; Thermo). Second derivative IR spectra were fitted simultaneously with the original IR spectra.

### Electronic nose analysis

The distinction of odor profiles of the MRPs was carried out based on an E-nose equipped with 14 metal oxide sensors (Intelligent Sensory Laboratory of Zhejiang Gongshang University, China). The sensor signal was brought down to zero by cleaning the sensor array with processed pure air (carrier gas) before analysis. For analysis, the MRPs solutions (10 mL) were transferred to a 30-mL glass vial and capped with a silicon rubber cap. Then, the sensor arrays were used to sample the volatile gas in headspace by a sampling needle after incubation at 50 ℃ for 20 min. The analysis parameters were as follows: the acquisition duration of the analyzer was 20 s with a rate of 150 mL/min, and a syringe flushing time was 120 s (Huang et al. 2011). All the samples were measured in triplicate. Principal component analysis (PCA) plot of the E-nose system was applied to analyze the results.

### Electronic tongue analysis

Taste characteristics of MRPs were detected using an electronic tongue equipped with 6 metal oxide sensors (Intelligent Sensory Laboratory of Zhejiang Gongshang University, China). The electrodes were immersed in distilled water and then dipped into the sample solutions for 30 s to proceed taste analysis. Each sample was measured in sextuplicate. PCA plot of the E-tongue system was applied to analyze the results.

### Volatile components analysis using gas chromatography–mass spectrometry (GC–MS)

Volatile extraction by headspace solid-phase microextraction (HS-SPME) was conducted according to Chiang et al. with some modifications (Chiang et al. 2019). Five milliliters of MRPs were placed in 20 mL flat bottom headspace vials and sealed with PTFE-coated silicone septa screw caps, with 1,2-dichlorobenzene in methanol (50 μL, 5 μg/mL) as an internal standard. Samples were then separated in a DB-5MS capillary column (60 m × 0.32 mm × 1 µm internal diameter, 1 mm in film thickness; J&W Scientific Inc., Folsom, CA, USA). The vial was kept at 55 °C in a thermal block for 10 min to equilibrate. After that, an SPME fiber was exposed to the headspace and maintained for 40 min.

After extraction, the compounds absorbed by the fiber were inserted into the GC (TRACE1300ISQQD, Thermo Fisher Scientific, Massachusetts, USA) injection port set at 240 °C for 5 min with a splitless injection mode. Helium was used as carrier gas with a flow rate of 1 mL/min. The GC oven temperature program for the SPME procedure: 40 °C for 3 min, 40–100 °C at 3 °C/min, 100–150 °C at 2 °C/min, 150–240 °C at 8 °C/min, and final temperature holding for 5 min.

The mass spectrometer was carried out with electron impact mode at 70 eV and the ion source temperature was set at 250 °C. The detector voltage was 350 V and the scan range of 35–500 amu. The NIST 08 and the literature database were used to identify volatile compounds, and the relative content was computed by the peak area.

### Analysis using gas chromatography–ion mobility spectrometry (GC–IMS)

The volatile compounds of MRPs were measured by a GC–IMS equipment (FlavourSpec^®^) from Gesellschaft für Analytische Sensorysteme mbH (G.A.S., Dortmund, Germany). Briefly, 2 g of sample was placed in a 20-mL vial and incubated at 55 °C for 20 min. After that, 500 μL of sample headspace was injected automatically by a heated syringe (85 °C) into the heated injector (85 °C) of the GC–IMS instrument. Then the samples were transferred into a WAX capillary column (30 m × 0.53 mm, 1 μm film thickness) (RESTEK, Bellefonte, US) by nitrogen (99.99%) at a programmed flow as follows: initially 2.0 mL/min for 10 min, 10 mL/min for 10 min and eventually 100 mL/min for 20 min. The ions of analytes ionized were directed to the drift tube with a constant temperature of 45 °C and the drift gas (nitrogen gas, 99.99% purity) was set at 150 mL/min. The final results were the averages of three replicates (Li et al. 2021).

Data were acquired by the integrated computer within the instrument and processed using the software Laboratory Analytical Viewer (LAV) from G.A.S. Compound identification was based on NIST 2014 (National Institute of Standards and Technology, Gaithersburg, MD, USA) and IMS mass spectral databases.

### Sensory evaluation

The quantitative descriptive sensory analysis was applied for evaluating MRPs by a well-trained panel consisting of 10 members aged between 23 and 28 from our lab according to previous method (Ogasawara et al. 2006). All panelists had previous experience with sensory evaluation over 6 months and had sensory experience with MRP samples over 3 months.

In this study, eight flavor characteristics including meaty, caramel, umami, salty, bitter, mouthfulness, continuity (long-lasting taste development), and total acceptance were used for the descriptive analysis. The specific method was used to dissolve MRPs solution (0.5%, w/v) in a mixture composed of 1.0% (w/v) sodium glutamate and 0.5% (w/v) NaCl. The evaluation was performed using a 1–10 interval scale (0 = none, 10 = extremely strong). The assessment of samples was done in triplicate by each panelist, and the average of all the panelists was calculated for each sample.

### Statistical analysis

All the experiments were performed at least triplicate determinations. SPSS 20.0 software (SPSS, Inc., Chicago, IL, USA) was used for data analysis. One-way ANOVA and Duncan’s test method were used to test whether there was significant difference in the mean value of each parameter. The data were expressed as mean ± standard deviation (*p* < 0.05). Mantel tests were performed to analyze the correlations between organoleptic characteristics and physicochemical properties using R software (version 4.1.1) with the vegan package, and visualized by the corrplot package.

## Results and discussion

### Intermediate products and browning intensity

The uncolored intermediate compounds, as an index of the formation of intermediate flavor products, are important precursors of the Maillard reaction which usually display a characteristic absorption spectrum with band maxima at 294 nm. While the brown polymers in more advanced stages of the Maillard reaction usually display a characteristic absorption spectrum at 420 nm (Rizzi 2010).

**Fig. 1:**
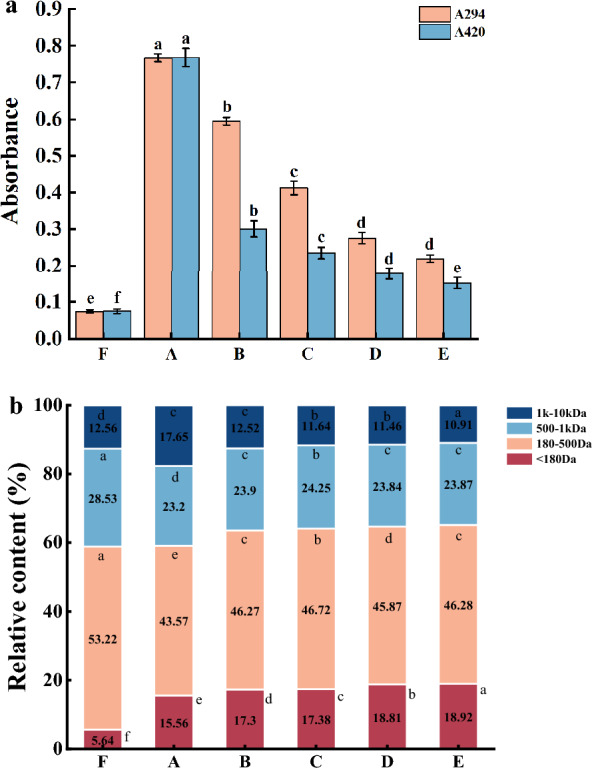
Effect of cysteine on the intermediate products, browning intensity (**a**), and molecular weight distribution (**b**) of MRPs. Different lowercase letters mean significant differences (*P* < 0.05) among different treatments. F: samples without cysteine addition and heating, A: Maillard reaction products without cysteine addition, B: Maillard reaction products with 0.25% cysteine addition, C: Maillard reaction products with 0.5% cysteine addition, D: Maillard reaction products with 0.75% cysteine addition, E: Maillard reaction products with 1.0% cysteine addition

As presented in Fig. 1a, the absorbance of the MRPs solutions both at 294 nm and 420 nm significantly (*p* < 0.05) increased after the Maillard reaction, indicating the formation of intermediate and final products. The highest values of both A_294_ and A_420_ were found in sample A (without cysteine addition), and then both decreased gradually with the increasing cysteine concentration, suggesting the inhibitory effect of cysteine on the formation of browning and brown compounds (melanoidins) (Yu et al. 2012). This may be due to the sulfhydryl functional groups in cysteine. Also, the intermediates formed in the initial stage of the reaction often account for a large proportion, which can inhibit the Maillard reaction and reduce browning intensity (Jin et al. 2018). The changes in browning intensity confirmed that cysteine is an important factor affecting the browning degree of Maillard reaction.

### Free amino acid content

FAAs are not only important flavor substances, but also flavor precursors involved in the Maillard reaction. The composition of free amino acids of samples A–F is compared in Table 1. The content of total FAA in MRPs decreased firstly and then increased with the increase of cysteine concentration. The decrease in FAAs content is mainly related to the cross-linking between xylose and FAAs residues or the combination with amino acids to form non-volatile compounds during heating (Lan et al. 2010). However, the increase in total FAA content indicated that the cross-linking between sugars and amino acids could be inhibited by the addition of cysteine.

**Table 1:** Change in free amino acid contents as a function of cysteine

Items		Free amino acid contents (g/100 g)					
		F	A	B	C	D	E
1	Asp	0.034 ± 0.01^a^	0.0485 ± 0.05^a^	0.06 ± 0.03^a^	0.064 ± 0.01^a^	0.050 ± 0.02^a^	0.050 ± 0.02^a^
2	Glu	0.156 ± 0.013^c^	0.236 ± 0.03^ab^	0.245 ± 0.02^a^	0.247 ± 0.01^a^	0.237 ± 0.03^a^	0.227 ± 0.03^b^
	TUFAA	0.190 ± 0.012^d^	0.284 ± 0.08^bc^	0.305 ± 0.05^a^	0.311 ± 0.02^a^	0.287 ± 0.01^ab^	0.276 ± 0.05^c^
1	Thr	0.030 ± 0.03^b^	0.10 ± 0.04^a^	0.096 ± 0.02^a^	0.099 ± 0.01^a^	0.096 ± 0.02^a^	0.095 ± 0.02^a^
2	Ser	0.020 ± 0.03^d^	0.164 ± 0.04^b^	0.155 ± 0.002^a^	0.171 ± 0.001^bc^	0.159 ± 0.004^c^	0.156 ± 0.002^c^
3	Pro	0.145 ± 0.05^e^	0.154 ± 0.01^e^	0.230 ± 0.02^d^	0.364 ± 0.01^c^	0.586 ± 0.04^b^	0.809 ± 0.04^a^
4	Gly	1.33 ± 0.02^ab^	1.312 ± 0.03^ab^	1.362 ± 0.016^a^	1.350 ± 0.03^a^	1.351 ± 0.014^a^	1.272 ± 0.002^b^
5	Ala	0.250 ± 0.005^a^	0.134 ± 0.002^b^	0.114 ± 0.002^c^	0.105 ± 0.02^d^	0.108 ± 0.003^ cd^	0.092 ± 0.004^e^
	TSFAA	1.776 ± 0.02^f^	1.863 ± 0.03^e^	1.956 ± 0.02^d^	2.085 ± 0.03^c^	2.299 ± 0.008^b^	2.424 ± 0.005^a^
1	Val	0.214 ± 0.002^a^	0.121 ± 0.009^b^	0.122 ± 0.002^b^	0.115 ± 0.004^b^	0.111 ± 0.0053^b^	0.112 ± 0.001^b^
2	Ile	0.217 ± 0.01^b^	0.296 ± 0.006^a^	0.208 ± 0.01^b^	0.206 ± 0.009^b^	0.172 ± 0.001^c^	0.16 ± 0.002^c^
3	Leu	0.52 ± 0.004^a^	0.355 ± 0.02^bc^	0.3315 ± 0.01^c^	0.339 ± 0.009^bc^	0.339 ± 0.001^bc^	0.366 ± 0.002^b^
4	Tyr	0.743 ± 0.01^a^	0.517 ± 0.03^b^	0.550 ± 0.002^b^	0.519 ± 0.01^b^	0.447 ± 0.009^c^	0.511 ± 0.007^b^
5	Phe	0.582 ± 0.01^a^	0.451 ± 0.002^b^	0.462 ± 0.004^b^	0.480 ± 0.002^b^	0.520 ± 0.002^ab^	0.477 ± 0.07^b^
6	His	0.047 ± 0.004^c^	0.103 ± 0.001^a^	0.096 ± 0.001^a^	0.081 ± 0.002^b^	0.083 ± 0.001^b^	0.096 ± 0.01^a^
7	Lys	0.151 ± 0.002^d^	0.212 ± 0.004^b^	0.24 ± 0.001^a^	0.245 ± 0.002^a^	0.145 ± 0.001^e^	0.180 ± 0.001^c^
8	Arg	0.019 ± 0.002^f^	0.135 ± 0.002^e^	0.144 ± 0.001^d^	0.167 ± 0.003^c^	0.181 ± 0.001^b^	0.19 ± 0.001^a^
	TBFAA	2.450 ± 0.07^a^	2.186 ± 0.07^b^	2.152 ± 0.002^b^	2.150 ± 0.01^bc^	1.997 ± 0.009^c^	2.100 ± 0.07^bc^
1	Met	0.282 ± 0.001^a^	0.157 ± 0.002^b^	0.146 ± 0.001^b^	0.146 ± 0.002^b^	0.148 ± 0.001^b^	0.149 ± 0.01^b^
2	Cystine	0.219 ± 0.007^b^	0.19 ± 0.005^c^	0.231 ± 0.002^a^	0.255 ± 0.002^a^	0.214 ± 0.004^b^	0.215 ± 0.002^b^
	TFAA	4.958 ± 0.04^e^	4.680 ± 0.10^de^	4.790 ± 0.03^d^	4.926 ± 0.04^c^	4.943 ± 0.005^b^	5.164 ± 0.10^a^

F: samples without cysteine addition and heating, A: Maillard reaction products without cysteine addition, B: Maillard reaction products with 0.25% cysteine addition, C: Maillard reaction products with 0.5% cysteine addition, D: Maillard reaction products with 0.75% cysteine addition, E: Maillard reaction products with 1.0% cysteine addition

*TUFAA* total umami free amino acids, *TSFAA* total sweet free amino acids, *TBFAA* total bitter free amino acids

Results are mean ± standard deviation (*n* = 3). Values with different superscript lowercase letters in the same column mean differ significantly (*p* < 0.05)

Amino acids showed significant impact on the sensory characteristics of MRPs including two umami amino acids (Glu and Asp), six bitter amino acids (Tyr, Ile, Leu, Val, Phe, and Lys), five sweet amino acids (Thr, Ser, Pro, Gly, and Ala), and two sulfur-containing amino acids (Cystine and Met). The content of umami amino acids firstly increased and then decreased with the increase of cysteine concentration, and sample C had the highest concentration of 0.311 mg/g. The content of sweet FAA showed similar trend, with sample E had the highest concentration of 2.424 mg/g. In particular, the content of proline kept increasing significantly from 0.145 to 0.809 mg/g. The increased content of umami and sweet amino acids might be due to the degradation of peptides (Cerny and Davidek 2004), indicating that cysteine might promote the degradation of peptides.

In contrast, the content of bitter FAA in all MRPs decreased with the increase of cysteine concentration, suggesting that cysteine could promote the reduction of bitter FAAs content, thereby reducing the bitterness and improving the overall acceptability of the final product of the reaction. The reduction of sulfur-containing FAA might be related to the thermal degradation and Strecker degradation in the Maillard reaction, which is involved in the generation of volatile compounds and contributes greatly to the strong meaty aroma of the samples (Shen et al. 2021).

### Molecular weight (MW) distribution

The MW distribution of the MRPs was significantly affected by cysteine addition (Fig. 1b). Compared with sample F, the contents of peptide fractions ranging 180 Da–500 Da and 500 Da–1 kDa of sample A were all significantly reduced by 9.65% and 5.33%, respectively, after the Maillard reaction, while the contents of peptides fractions below 180 Da and 1 kDa–10 kDa were significantly increased by 9.92% and 5.09%, respectively. This suggested that peptide degradation and peptide cross-linking occurred simultaneously in the Maillard reaction, affirming that the increase in the percentage of high molecular weight peptides in MRPs might be attributed to the cross-linking of small peptides. These results are consistent with the findings of Yu et al. (Yu et al. 2018). And peptide fraction ranging 180 Da–1 kDa might be the main participants in this cross-linking reaction along with high reaction activity.

However, low MW peptides below 500 Da in MRPs gradually increased from 59.13 to 65.2%, while peptide fractions ranging 1 kDa–10 kDa gradually decreased from 17.65 to 10.91% with the increasing cysteine concentration. The result indicated that the addition of cysteine could significantly inhibit the cross-linking of peptides, and could also accelerate the degradation rate of peptides which were consistent with previous findings (Wei et al. 2019).

### Fourier transform infrared spectroscopy (FTIR)

**Fig. 2:**
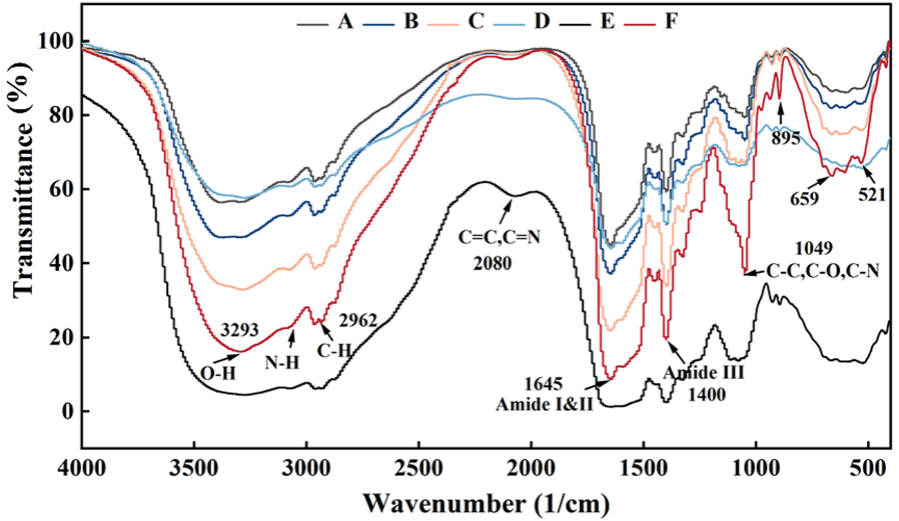
Effect of cysteine on the FTIR spectra of MRPs. Different lowercase letters mean significant differences (*P* < 0.05) among different treatments. F: samples without cysteine addition and heating, A: Maillard reaction products without cysteine addition, B: Maillard reaction products with 0.25% cysteine addition, C: Maillard reaction products with 0.5% cysteine addition, D: Maillard reaction products with 0.75% cysteine addition, E: Maillard reaction products with 1.0% cysteine addition

FTIR spectroscopy revealed the influence of cysteine on the structure of MRPs. As shown in Fig. 2, the IR spectra of MRPs in samples A–E were slightly different compared to the unheated counterpart (F), indicating that the Maillard reaction process had considerably affected the peptide structure. After Maillard reaction, the peak intensities of MRPs decreased in amide A, B, I, II, amide III, C-O, and 500–700 cm^−1^ regions, which indicated cross-linking interaction between sugars and amino acids/polypeptides.

The transmittance intensity of amides A, I, II, and III gradually increased with the increase of cysteine concentration, with the peak at 659 cm^−1^ gradually disappeared, especially the peak intensity at 1049 cm^−1^. Considering that the formation of the bonds (C-O, N–H, and C-N) of the amide groups associated with MRPs (e.g., Amadori compounds, Schiff bases, and pyrazines) are absorbed in this range (Nooshkam and Madadlou 2016), it might indicate that the addition of cysteine inhibited the cross-linking and this result was consistent with the MW distribution analysis result.

### Electronic nose and electronic tongue analysis

**Fig. 3:**
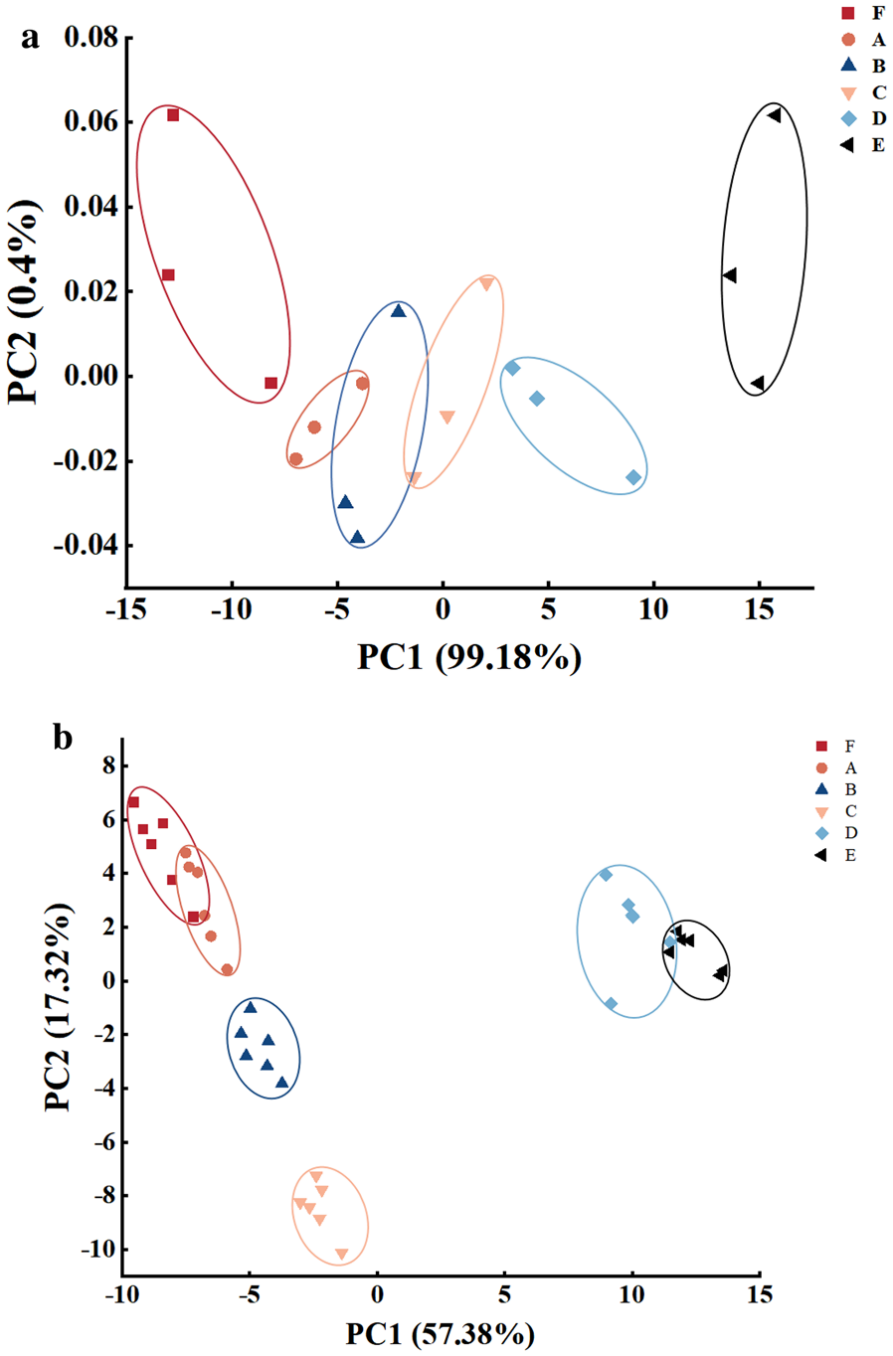
Principal component analysis (PCA) of electronic nose (**a**) and electronic tongue (**b**) sensor data of unheated sample (F) and MRPs with different amounts of cysteine added (A, B, C, D, and E). F: samples without cysteine addition and heating, A: Maillard reaction products without cysteine addition, B: Maillard reaction products with 0.25% cysteine addition, C: Maillard reaction products with 0.5% cysteine addition, D: Maillard reaction products with 0.75% cysteine addition, E: Maillard reaction products with 1.0% cysteine addition

PCA plot of electronic nose sensor data showed that principal components PC1 and PC2 accounted for 99.48% and 0.30% of the total variance, respectively, and the total contribution rate is 99.78% (Fig. 3a), indicating that the principal components can reflect the indicator information well. The regions of samples F and A–E were clearly separated, illustrating a significant difference in the aroma characteristics; while A, B, and C showed similar aroma characteristics. The distances between B, C, D, E, and A increased gradually, indicating that the aroma difference became larger with the increasing cysteine concentration. The distance between E and other samples (F–D) was larger relatively, which might be due to the larger change in aroma when the cysteine concentration is greater than 0.5%.

Principal components PC1 and PC2 in the PCA plot of electronic tongue sensor data accounted for 57.38% and 17.32% of the total variance, respectively (Fig. 3b). The taste profile of each group basically separated from each other, indicating significantly different flavor characteristics among samples with different amounts of cysteine added. The taste changed greatly after the Maillard reaction compared with sample F. Overlapping among samples F and A, D and E indicated relationship in their taste profile among each other. In addition, the taste profiles of sample A and other samples were graphically separated from each other, demonstrating that there is a significant difference in taste among groups with and without cysteine. The distances between A and E gradually became farther, indicating that the higher the cysteine concentration, the greater the difference. Moreover, the farthest distance was found among D, E and C, and this might be due to the large change in taste caused by the high cysteine concentration (> 0.5%).

### Volatile components analysis using GC–MS

Based on the GC–MS results, 88 volatile compounds significantly contributing to the flavor properties of MRPs can be classified into four categories (Table 2), including 7 furans, 12 sulfur-containing compounds (4 aliphatic sulfur compounds, 3 thiols, 4 thiophenes, and 1 thiazole), 18 nitrogen-containing compounds (13 pyrazines, 1 pyridine, 2 pyrroles, 1 pyrimidine, 1 imidazole), and 53 oxygen-containing compounds (24 aldehydes, 9 ketones, 8 alcohols, 3 acids, 2 esters, and 5 phenols) (Eric et al. 2014).

**Table 2:** Changes in volatile compounds as a function of cysteine

No.	CAS	Compounds	F	A	B	C	D	E	Odor description
		Furans		179.9 ± 7.21	36.62 ± 2.54	13.39 ± 1.25	15.40 ± 1.03	8.13 ± 0.92	
1	534–22-5	2-Methylfuran	ND	13.50 ± 1.21^a^	ND	ND	ND	ND	Chocolate, coffee, nut
2	98–01-1	Furfural-M	ND	121.7 ± 10.2^a^	32.33 ± 2.8^b^	9.54 ± 0.83^c^	10.94 ± 0.24^c^	3.30 ± 0.12^c^	Almond
3	1192–62-7	2-Acetylfuran	ND	3.70 ± 0.28^b^	2.89 ± 0.53^d^	3.55 ± 0.12^c^	4.46 ± 0.41^a^	3.37 ± 0.22^c^	Almond, nutty, milk,
4	3194–15-8	1-Propanone,1-(2-furanyl)-	ND	2.80 ± 0.11^a^	0.72 ± 0.31^b^	0.3 ± 0.05^c^	ND	ND	Sulfurous
5	13679–41-9	3-Phenylfuran	ND	2.31 ± 0.16^a^	0.67 ± 0.07^b^	ND	ND	ND	
6	19377–82-3	2-Furanmethanamine,N-(2-furanylmethylene)-	ND	35.89 ± 2.42^a^	ND	ND	ND	ND	
7	3777–69-3	2-Amylfuran	ND	ND	ND	ND	ND	1.45 ± 0.23^a^	Grass
		Sulfur-containing compound	ND	17.34 ± 1.25	25.05 ± 2.46	62.27 ± 5.31	298.50 ± 21.43	190.14 ± 11.34	
8	624–92-0	Dimethyl disulfide	ND	5.56 ± 0.2^a^	ND	ND	ND	ND	Sulfurous, vegetable, onion
9	3658–80-8	Dimethyl trisulfide	ND	1.94 ± 0.1^a^	ND	ND	ND	ND	Sulfurous, cooked onion, meaty
10	3268–49-3	3-(Methylthio) propionaldehyde	ND	8.81 ± 0.67^b^	12.09 ± 0.7^a^	4.34 ± 0.13^c^	1.59 ± 0.04^d^	1.55 ± 0.03^d^	Meaty, cooked onion, seafood
11	24295–03-2	2-Acetylthiazole	ND	1.03 ± 0.02^b^	3.26 ± 0.12^b^	14.35 ± 0.78^a^	1.36 ± 0.06^ab^	1.26 ± 0.05^b^	Beef, nutty, popcorn, roasted peanuts
12	28588–74-1	2-Methyl-3-furanthiol	ND	ND	4.31 ± 0.25^d^	18.16 ± 1.23^c^	43.73 ± 3.52^b^	55.35 ± 3.87^a^	Meaty
13	932–95-6	2,5-Thiophenedicarboxaldehyde	ND	ND	3.98 ± 0.14^d^	16.79 ± 0.98^b^	32.23 ± 2.81^a^	14.87 ± 0.84^c^	
14	57500–00-2	Methyl furfuryl disulfide	ND	ND	1.41 ± 0.08^b^	2.21 ± 0.22^a^	ND	ND	Sulfury, coffee, roasted alliaceous, meaty
15	5834–16-2	3-Methyl-2-thiophene carboxaldehyde	ND	ND	ND	6.16 ± 0.73^b^	8.67 ± 0.35^a^	5.52 ± 0.43^c^	
16	98–03-3	2-Thenaldehyde	ND	ND	ND	0.26 ± 0.03^b^	1.14 ± 0.05^a^	ND	Bitter almonds
17	98–02-2	Furfuryl mercaptan	ND	ND	ND	ND	209.8 ± 12.41^a^	111.6 ± 8.3^b^	Sulfury, roasted, coffee, fatty
18	1639–04-9	2-Methyl-3-pentanethiol	ND	ND	ND	ND	1.24 ± 0.15^a^	ND	
19	554–14-3	2-Methylthiophene	ND	ND	ND	ND	ND	4.87 ± 0.28^a^	Sulfurous, alliaceous onion, green
		Nitrogen-containing compounds	3.1 ± 0.21	1119.0 ± 32.83	351.00 ± 18.76	314.99 ± 23.85	49.14 ± 2.96	40.60 ± 3.98	
20	290–37-9	Pyrazine	ND	ND	3.93 ± 0.29^a^	ND	ND	ND	Sweet corn
21	5910–89-4	2,3-Dimethyl pyrazine	ND	0.69 ± 0.16^a^	ND	ND	ND	ND	Nutty, peanut, coffee, walnut caramel
22	14667–55-1	2,3,5-Trimethyl Pyrazine	ND	47.62 ± 2.17^a^	ND	ND	ND	ND	Nutty, potato, roasted peanut
23	13360–65-1	3-Ethyl-2,5-dimethyl- Pyrazine	ND	34.22 ± 2.29^a^	ND	ND	ND	ND	Potato, cocoa, roasted nutty
24	91010–41-2	2-Methyl-6-(3-methyl-butyl)-pyrazine	ND	1.33 ± 0.32^a^	ND	ND	ND	ND	
25	56617–70-0	3,5-Dimethyl-2-(2-methylbutyl)- (9CI) pyrazine	ND	10.22 ± 1.37^a^	ND	ND	ND	ND	
26	18433–98-2	2,5-Dimethyl-3-(3-methylbutyl)- Pyrazine	ND	104.3 ± 5.6^a^	21.77 ± 1.86^b^	ND	ND	ND	Fruity
27	18138–03-9	2-Propylpyrazine	ND	ND	17.51 ± 1.25^a^	6.85 ± 0.53^b^	ND	ND	Nutty
28	29460–92-2	2-(2-Methylpropyl)-pyrazine	ND	ND	1.10 ± 0.33^b^	4.33 ± 0.15^a^	4.45 ± 0.47^a^	1.13 ± 0.23^b^	
29	13925–07-0	2-Ethyl-3,5-dimethyl- Pyrazine	ND	ND	6.57 ± 0.26^a^	2.38 ± 0.37^b^	ND	ND	Almonds, roasted nuts, coffee
30	29461–03-8	2-Methyl-5-propyl-pyrazine	ND	ND	ND	ND	ND	5.46 ± 0.48^a^	
31	109–08-0	2-Methyl pyrazine	ND	54.86 ± 3.64^c^	78.98 ± 5.43^b^	88.70 ± 9.25^a^	44.69 ± 3.92^d^	32.75 ± 2.43^e^	Nutty, cocoa, chocolate, peanut
32	123–32-0	2,5-Dimethyl pyrazine	3.1 ± 0.43^d^	851.9 ± 24.32^a^	216.1 ± 13.52^b^	202.1 ± 15.26^c^	ND	ND	Cocoa, roasted nuts, roast beef
33	1438–94-4	1-Furfurylpyrrole	ND	6.99 ± 0.45^a^	ND	ND	ND	ND	Green, fruity, coffee, vegetable
34	616–43-3	3-Methyl-1H-pyrrole	ND	ND	ND	ND	ND	1.26 ± 0.05^a^	
35	274–40-8	Indolizine	ND	ND	5.09 ± 0.67^a^	3.13 ± 0.26^b^	ND	ND	
36	289–95-2	Pyrimidine	ND	6.87 ± 0.53^a^	ND	7.22 ± 0.83^a^	ND	ND	
37	16975–71-6	2-Ethenyl-1-methylimidazole	ND	ND	ND	0.27 ± 0.03^a^	ND	ND	
The oxygen-containing compounds									
		Aldehydes	211 ± 14.68	136.43 ± 10.52	218.13 ± 14.89	275.40 ± 21.47	421.63 ± 32.58	280.11 ± 23.24	
38	110–62-3	Valeraldehyde	1.46 ± 0.45^a^	ND	ND	ND	ND	ND	
39	1860–39-5	5-Methylhexanal	3.03 ± 0.24^a^	ND	ND	ND	2.54 ± 0.12^b^	ND	
40	66–25-1	Hexanal	13.56 ± 2.11^b^	7.60 ± 0.52^d^	8.44 ± 0.65^d^	11.83 ± 1.58^c^	15.44 ± 1.26^a^	12.55 ± 1.32^c^	Green, fatty, grass, fruity sweaty
41	111–71-7	Heptaldehyde	8.11 ± 0.65^a^	4.25 ± 0.34^c^	5.12 ± 0.59^b^	5.86 ± 0.42^b^	8.78 ± 0.64^a^	7.71 ± 0.84^a^	Fruity
42	57266–86-1	2-Heptenal, (2Z)-	2.39 ± 0.13^a^	ND	ND	ND	ND	2.12 ± 0.19^a^	
43	100–52-7	Benzaldehyde	32.04 ± 3.42^d^	27.30 ± 1.42^e^	69.80 ± 5.23^c^	108.1 ± 9.75^b^	138.0 ± 12.3^a^	70.15 ± 6.75^c^	Bitter almond, cherry
44	4313–03-5	2,4-Heptadienal, (E,E)-	8.78 ± 0.62^a^	ND	ND	ND	6.13 ± 0.39^b^	4.44 ± 0.22^c^	Fatty, vegetable
45	124–13-0	Octanal	6.56 ± 0.34^d^	ND	ND	9.76 ± 0.45^b^	11.36 ± 1.36^a^	7.96 ± 0.81^c^	Orange, fatty
46	122–78-1	Benzeneacetaldehyde	5.35 ± 0.67^d^	13.49 ± 0.95^a^	12.21 ± 0.78^ab^	11.26 ± 1.33^b^	7.12 ± 0.84^c^	4.94 ± 0.23^d^	Rose, fruity
47	2548–87-0	(E)-2-Octenal	4.19 ± 0.44 ^b^	ND	ND	ND	ND	6.44 ± 0.73^a^	Fresh cucumber, fatty, banana
48	124–19-6	Nonanal	48.10 ± 3.56^c^	24.33 ± 1.76^e^	37.44 ± 1.75^d^	51.35 ± 4.58^c^	96.64 ± 10.41^a^	75.29 ± 6.54^b^	Rose, fatty
49	112–31-2	Decanal	10.04 ± 1.24^d^	6.33 ± 0.34^e^	10.76 ± 0.89^d^	14.23 ± 1.25^c^	18.15 ± 1.64^b^	23.11 ± 3.21^a^	fatty, fruit
50	15764–16-6	2,4-Dimethylbenzaldehyde	5.70 ± 0.64^a^	ND	ND	ND	ND	ND	Bitter almond, sweet
51	2497–25-8	(Z)-2-Decen-1-al	9.32 ± 1.27^a^	3.37 ± 0.23^c^	2.64 ± 0.18^d^	3.64 ± 0.27^c^	5.71 ± 0.48^b^	5.37 ± 0.46^b^	Butter almond
52	28785–06-0	Benzaldehyde, 4-propyl-	19.79 ± 1.65^a^	13.75 ± 1.54^c^	16.53 ± 1.48^b^	ND	ND	ND	
53	112–44-7	Undecanal	3.92 ± 0.26^b^	ND	3.34 ± 0.45^b^	2.54 ± 0.16^c^	4.57 ± 0.22^a^	4.41 ± 0.50^a^	Fruity, rose, flower,
54	25152–84-5	(E,E)-2,4-Decadienal	9.92 ± 1.01^a^	ND	ND	ND	ND	ND	Cucumber, citrus, nutty
55	2463–77-6	Undecenal	15.72 ± 1.29^a^	4.33 ± 0.39^c^	4.84 ± 0.26^c^	3.53 ± 0.16^d^	7.15 ± 0.54^b^	7.02 ± 0.62^b^	Grass, fatty, citrus
56	112–54-9	Dodecyl aldehyde	3.94 ± 0.08^c^	ND	ND	2.83 ± 0.13^d^	5.02 ± 0.39^a^	4.39 ± 0.23^b^	Soapy, aldehydic, green floral
57	590–86-3	3-Methylbutyraldehyde	ND	5.27 ± 0.36^a^	4.15 ± 0.14^b^	2.99 ± 0.31^c^	ND	ND	
58	2548–87-0	(E)-2-Octenal	ND	0.40 ± 0.12^d^	0.61 ± 0.03^c^	0.82 ± 0.04^b^	2.32 ± 0.18^a^	ND	Meaty, fatty, chicken
59	56599–95-2	2-Bromo octadecanal	ND	3.52 ± 0.21^d^	10.44 ± 0.92^c^	4.79 ± 0.26^d^	17.09 ± 0.95^b^	44.21 ± 1.58^a^	
60	21662–16-8	(E,E)-2,4-Dodecadienal	ND	3.42 ± 0.24^b^	0.86 ± 0.04^c^	0.83 ± 0.06^c^	6.70 ± 0.03^a^	ND	
61	638–66-4	Octadecanal	ND	19.08 ± 1.28^d^	30.96 ± 3.67^c^	41.04 ± 3.95^b^	68.95 ± 5.83^a^	ND	
		Alcohols	14.36 ± 1.20	10.02 ± 1.07	6.32 ± 0.52	5.02 ± 0.43	30.63 ± 0.29	19.13 ± 1.12	
62	89182–08-1	1-Cyclobutene-1-methanol	5.40 ± 0.24^a^	1.16 ± 0.08^c^	ND	1.81 ± 0.11^b^	ND	ND	
63	4706–89-2	2-Tetradecanol	1.42 ± 0.13^a^	ND	ND	ND	ND	ND	
64	2490–48-4	1-Hexadecanol,2-methyl-	7.53 ± 0.34^a^	3.30 ± 0.24^b^	2.01 ± 0.12^c^	1.07 ± 0.08^e^	3.68 ± 0.26^b^	1.66 ± 0.09^d^	
65	111–28-4	2,4-Hexadien-1-ol	ND	ND	4.31 ± 0.32^a^	2.14 ± 0.15^c^	1.96 ± 0.15^c^	2.53 ± 0.18^b^	
66	6261–22-9	2-Pentyn-1-ol	ND	ND	ND	ND	3.61 ± 0.28^a^	ND	
67	6712–79-4	Isopinocarveol	ND	ND	ND	ND	8.25 ± 0.69	10.07 ± 1.25	
68	694–29-1	cis-1-Cyclopentene-3,4-diol	ND	ND	ND	ND	13.12 ± 1.84^a^	ND	
69	56554–77-9	13-Heptadecyn-1-ol	ND	ND	ND	ND	ND	4.86 ± 0.35^a^	
		Ketones	ND	16.59 ± 3.04	18.64 ± 2.36	35.59 ± 3.29	61.51 ± 7.35	73.25 ± 5.42	
70	51004–21-8	2-Butanone,4-cyclopentylidene-	ND	1.50 ± 0.09^a^	ND	ND	ND	ND	
71	3796–70-1	Geranylacetone	ND	3.52 ± 0.26^d^	4.84 ± 0.32^c^	6.42 ± 0.49^b^	10.42 ± 1.04^a^	4.80 ± 0.33^c^	Grass, rose
72	111–13-7	2-Octanone	ND	1.99 ± 0.12^c^	2.76 ± 0.26^a^	2.41 ± 0.22^b^	2.93 ± 0.03^a^	2.49 ± 0.10^b^	Apple, grass, floral
73	116–09-6	Hydroxyacetone	ND	9.59 ± 1.01^b^	9.80 ± 2.30^b^	17.79 ± 2.54^a^	ND	ND	
74	110–43-0	2-Heptanone	ND	ND	1.24 ± 0.05^d^	1.56 ± 0.1^c^	2.45 ± 0.02^b^	3.48 ± 0.23^a^	Fruity, coconut, woody
75	13125–74-1	5,9-Dodecadien-1-one,6,10-dimethyl-,(E,E))-	ND	ND	ND	4.68 ± 0.23^b^	5.24 ± 0.45^a^	4.38 ± 0.52^b^	
76	24653–75-6	2-Propanone,1-mercapto- (8CI,9CI)	ND	ND	ND	2.01 ± 0.28^c^	10.94 ± 1.95^b^	14.28 ± 1.65^a^	
77	21856–89-3	6-Hydroxyhexan-2-one	ND	ND	ND	0.73 ± 0.02^a^	ND	ND	
78	67–64-1	Acetone	ND	ND	ND	ND	29.53 ± 3.01^b^	36.35 ± 3.57^a^	Apple, pear
79	107–87-9	2-Pentanone	ND	ND	ND	ND	ND	7.48 ± 0.44^a^	Fruity
		Esters	2.72 ± 0.14	2.84 ± 0.16	ND	ND	ND	4.46 ± 0.25	
80	623–42-7	Methyl butyrate	2.72 ± 0.14^b^	1.47 ± 0.05^b^	ND	ND	ND	4.46 ± 0.25^a^	Fruity, apple, banana
81	5331–43-1	Carbobenzoxyhydrazide	ND	1.37 ± 0.07^a^	ND	ND	ND	ND	
		Acids	ND	33.25 ± 4.23	55.38 ± 4.68	30.72 ± 3.28	39.28 ± 2.37	1.05 ± 0.01	
82	64–19-7	Acetic acid glacial	ND	33.25 ± 4.23^a^	22.74 ± 2.36^b^	18.73 ± 2.1^c^	ND	ND	Sharp pungent sour vinegar
83	57–10-3	Palmitic acid	ND	ND	32.64 ± 2.84^b^	12.00 ± 0.87^c^	39.28 ± 3.16^a^	ND	
84	2091–29-4	9-Hexadecenoic acid	ND	ND	ND	ND	ND	1.05 ± 0.09^a^	
		Phenol	25.76 ± 1.63	7.06 ± 0.64	9.93 ± 0.88	8.72 ± 0.24	18.77 ± 0.85	18.78 ± 1.21	
85	6712–79-4	Isopinocarveol	1.71 ± 0.12^a^	ND	ND	ND	ND	ND	
86	96–76-4	2,4-Di-tert-butylphenol	3.98 ± 0.43^d^	7.06 ± 0.82^b^	5.90 ± 0.66^c^	4.72 ± 0.13^d^	13.67 ± 0.22^a^	14.17 ± 0.11^a^	
87	128–37-0	Butylated hydroxytoluene	20.07 ± 2.24^a^	ND	4.03 ± 0.29^b^	4.00 ± 0.16^b^	ND	ND	
88	497–39-2	4,6-Di-tert-butyl-m-cresol	ND	ND	ND	ND	3.45 ± 0.42^b^	4.60 ± 0.53^a^	

F: samples without cysteine addition and heating, A: Maillard reaction products without cysteine addition, B: Maillard reaction products with 0.25% cysteine addition, C: Maillard reaction products with 0.5% cysteine addition, D: Maillard reaction products with 0.75% cysteine addition, E: Maillard reaction products with 1.0% cysteine addition

*ND* not detected

Results are mean ± standard deviation (*n* = 3). Values with different superscript lowercase letters in the same column mean differ significantly (*p* < 0.05)

#### Furans (non-S-containing)

Furans can be produced by sugar caramelization or carbohydrate degradation and play important roles in the overall sensory attributes of foods, especially the caramel-like flavor (Limacher et al. 2008). It was observed in the present study that the furan content decreased from 179.9 to 8.1 ng/g with the increasing cysteine concentration. Sample A showed the highest furan content of 179.9 ng/g compared with other MRPs, 2-methyl-furan and furfural were the most abundant furans, having aromas of cocoa, nuts, and coffee, and their concentrations sharply increased with the increasing cysteine concentration. These results showed that the addition of cysteine in the Maillard reaction system did not favor the formation of non-sulfur-containing furans.

#### Sulfur-containing compounds

The heterocyclic compounds usually contribute to the overall cooked meat aroma and provide savory, meaty, roast, and boiled flavors in different foods due to their low odor threshold and characteristic odor (Ji et al. 2020). These sulfur-containing heterocyclic compounds may be formed by the thermal degradation of cysteine and the interaction between carbonyl compounds and sulfur-containing amino acids (Zhu et al. 2018). In the present study, the relative content of sulfur-containing compounds was richer in MRPs and increased with the increasing cysteine concentration. Sulfur-substituted furans possess strong meat-like and roast aromas with low odor threshold values. 2-Methyl-3-furanthiol and furfuryl mercaptan were the most dominant sulfur-substituted furans, which were considered as major contributors to the meaty aroma in cooked beef. It has been reported that furfuryl mercaptan showed strong coffee scents, and 2-methyl-3-furanthiol presented roast meat or coffee aroma, especially being suitable as a flavor enhancer for grilled meats, enemas, and broths (Wang et al. 2012). But, 2-methyl-3-furanthiol was only detected in cysteine-added MRPs, and its relative content increased significantly from 4.31 to 55.35 ng/g with the increase of cysteine concentration. 3-(methylthio) propionaldehyde has a strong onion and meat-like aroma and has the aroma of broth and seafood at low concentration. Its concentration increased firstly and then decreased with the increase of cysteine concentration. Furfuryl mercaptan was only detected in sample D and E, which has a strong nutty, meaty and sulfuric taste, and the concentrations were highly reached to 209.79 and 111.59 ng/g, respectively.

Thiazoles which contribute to the burnt, roasty, and meat-like flavor of foods were also only detected in cysteine-added MRPs. Among them, 2,5-thiophenedicarboxaldehyde and 3-methyl-2-thiophene carboxaldehyde were the most dominant thiazoles, and their relative content increased firstly and then decreased with the increasing cysteine concentration. 2-Methyl thiophene has sulfur and roasted onion flavors and was only detected in group E. They have been suggested as being responsible for the mild sulfurous odor of cooked meat.

In the case of thiazoles, they contribute to the burnt, roasty, and meat-like flavor of foods. Only one type of thiazole was detected, 2-acetylthiazole has beef, popcorn, nut, and roasted peanut aroma, its relative content increased firstly and then decreased with the increasing cysteine concentration. These results all indicated that the addition of cysteine could increase the formation of sulfur-containing compounds such as thiols, thiophenes, and thiazoles, which may have a significant impact on meat flavor.

#### Nitrogen-containing compounds

Previous studies reported that the Maillard reaction play an important role in the formation of more important nitrogen-containing heterocyclic compounds such as pyrazines, pyridines, and pyrroles (Li et al. 2020). Pyrazines were the major nitrogen-containing compounds which contribute to the toasted, roasted, nutty, and burnt notes of cooked foods and their concentration sharply reduced from 1105.15 to 39.34 ng/g with the increasing cysteine concentration. 2-Methyl pyrazine and 2,5-dimethyl pyrazine were the most important nitrogen-containing substances, and their concentrations in group A reached 54.86 ng/g and 851 ng/g, respectively. 2,3,5-Trimethylpyrazine, pyrazine, and 2,5-dimethyl-3-(3-methyl butyl)-pyrazine were only detected in MRPs without cysteine addition. These results indicated that the addition of cysteine could not be conducive to the formation of nitrogen-containing compounds, especially pyrazines.

#### The oxygen-containing compounds

According to previous studies, the formation of most oxygen-containing compounds might be caused by lipid oxidation, and they have a much higher odor threshold than sulfur- and nitrogen-containing heterocyclic compounds from water-soluble precursors, providing meaty and roast flavor (Eric et al. 2013). Therefore, they would have no obvious effect on the flavor characteristic of MRPs.

However, aldehydes and ketones are important flavor precursors in Maillard reaction systems. The contents of ketones and phenols in MRPs were increased with the increasing cysteine concentration, and among, geranylacetone and 2-octanone have grassy and floral aromas. The contents of aldehydes and alcohols firstly increased and then decreased, which might be due to the inhibitory effect of excessive cysteine on the formation of reaction products. Nonanal, decanal, trans-2-octenal, benzaldehyde, and hexanal were the most abundant aldehydes with oily aromas. Benzaldehyde is the most used aromatic aldehyde with a cherry flavor, while hexanal is the main component of vegetables and fruits with a fruity flavor. These aldehydes can reduce the unpleasant odors and improve the aroma of foods.

In summary, these results demonstrated that the addition of cysteine favors the formation of sulfur-containing compounds, which might be due to the decomposition of cysteine at high temperatures. However, the addition of cysteine is not conducive to the formation of furans and nitrogen-containing compounds, which may be due to the competitive reaction of cysteine and glycine with xylose that can inhibit the conversion of xylose to furans. In addition, 0.25–0.75% range of cysteine enriched the types of MRPs which greatly contributed to the aroma of meat such as barbecue and beef, and weakened the burnt taste.

### Volatile components analysis using GC–IMS

**Fig. 4:**
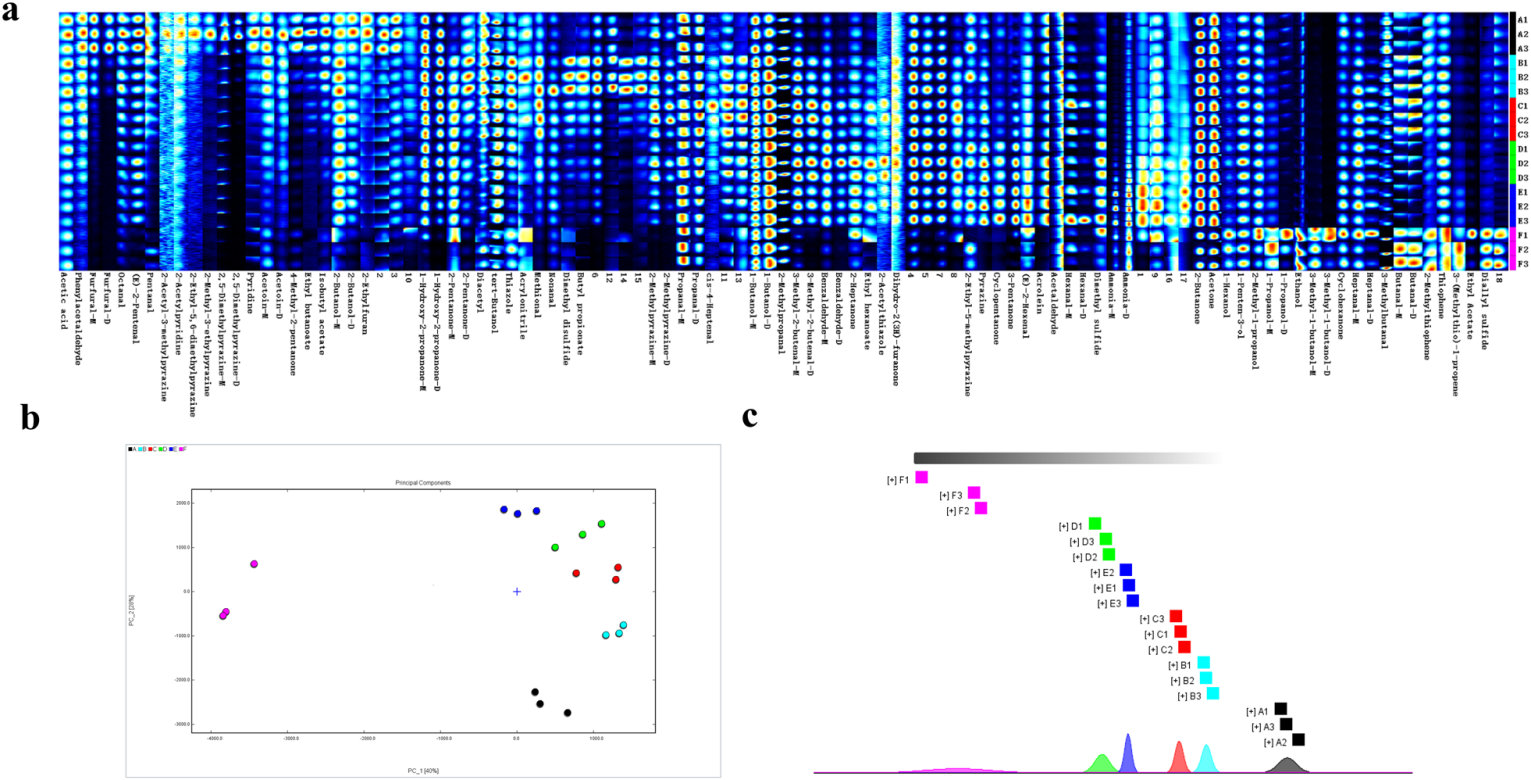
Effect of cysteine on the volatile components of MRPs by HS-GC–IMS analysis. VOCs fingerprint comparisons of unheated sample (F) and MRPs with different amounts of cysteine added (A, B, C, D, and E) (**a**). The redder the area is, the larger is the quantity of VOCs. Each row represents all the signals selected in a sample. Each column represents the signals of the same VOCs. (M) and (D) denote monomer and dimer, respectively. **b** and **c** Are principal component analysis and fingerprint similarity analysis, respectively, of VOCs in unheated sample (F) and MRPs added with different amounts of cysteine added. (A:0%, B:0.25, C:0.5%, D:0.75%, E:1%). MRPs: Maillard reaction products. VOCs: Volatile components

GC–IMS was further conducted to characterize the overall flavor properties and elucidate the differences of volatile components of the MRPs in five groups. A total of 60 effective organic volatiles including 1 furan, 9 sulfur-containing compounds, 9 nitrogen-containing compounds, 42 oxygen-containing compounds (15 aldehydes, 11 ketones, 10 alcohols, 5 esters, and 1 acid) and 2 other kinds of compounds (ammonia and acrylonitrile) were detected as shown in Fig. 4a.

The relative contents of five nitrogen-containing compounds (2,5-dimethylpyrazine-M, 2-methyl-3-ethyl pyrazine, 2-ethyl-5,6-dimethyl pyrazine, 2-acetylpyridin and 2-acetyl-3-methyl pyrazine) were the highest in group A (without cysteine) and decreased significantly with the increasing cysteine concentration, and the relative content of 2-methyl pyrazine firstly increased and then decreased with the increase of cysteine concentration.

In the case of sulfur-containing compounds, the relative contents of 2-acetylthiazole, dimethyl disulfide, methional, and thiazole were increased firstly and then decreased with the increasing cysteine concentration, and the relative content of dimethyl sulfide was only detected in groups with cysteine addition (B, C, D and E).

The changes of oxygenates showed obvious inconsistency. The content of some aldehydes including benzaldehyde, 3-methyl-2-butenal, (Z)-4-heptenal, propanal, nonanal, 3-methylthiopropanal, and ketones including n-butanol, 1-penten-3-ol, tert-butanol increased firstly and then decreased with the increase of cysteine concentration. The relative content of ketones including cyclohexanone, 3-pentanone, cyclopentanone and 2-heptanone increased with the increasing cysteine concentration, while the relative content of 2,3-butanedione, hydroxyacetone, 4-methyl-2-pentanone and 3-hydroxy-2-butanone decreased gradually.

These results demonstrated that the addition of cysteine favors the formation of sulfur-containing compounds and is not conducive to the formation of furans and nitrogen-containing compounds, but excessive cysteine concentration can also lead to a decrease in the relative content of some compounds, agreeing with the GC–MS results.

To highlight the differences in VOCs of MRPs added with different concentrations of cysteine, PCA and fingerprint similarity analysis (FSA) were performed according to the area signal intensities of VOCs as shown in Fig. 4b and c. According to the area intensities of VOCs, there were two principal components including PC1 and PC2 (Fig. 4b) with accumulative variance contribution rate accounted for 40% and 28%, respectively. MRPs of samples A, B, C, D, and E were arranged from bottom to upper in the figure. All the MRPs showed obvious differences and sample F and A were far away from other samples especially. FSA was applied to analyze the similarity of fingerprints of VOCs by calculating and comparing Euclidean distance. The larger the sample distance is in the figure, the more obvious the sample difference is, and the FSA results in Fig. 4c further confirmed the analytical conclusions of the PCA results in Fig. 4b.

### Sensory evaluation

**Fig. 5:**
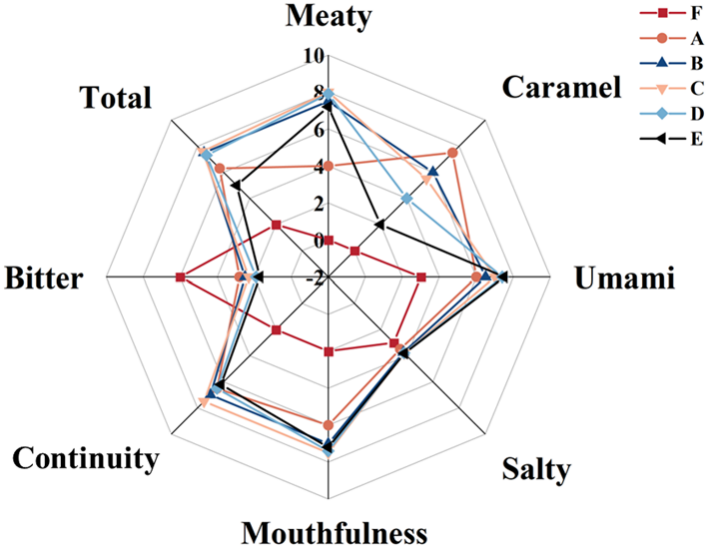
Effect of cysteine on the sensory properties of MRPs. F: samples without cysteine addition and heating, A: Maillard reaction products without cysteine addition, B: Maillard reaction products with 0.25% cysteine addition, C: Maillard reaction products with 0.5% cysteine addition, D: Maillard reaction products with 0.75% cysteine addition, E: Maillard reaction products with 1.0% cysteine addition

The sensory properties including meaty, caramel, umami, salty, bitter, mouthfulness, continuity, and total acceptance of the MRPs were markedly affected by cysteine addition (Fig. 5), especially in meaty and caramel flavors. After Maillard reaction, meaty, caramel, umami, salty, and overall acceptability of all MRPs were significantly increased, while the bitterness was significantly decreased. Previous studies also found that Maillard reaction increased kokumi and umami effect (Wakamatsu et al. 2016).

Comparatively, sample A obtained the highest caramel odor, possibly because they were rich in pyrazines and furans, and caramel flavor decreased with the increasing cysteine concentration. The meaty aroma increased with the increase of cysteine concentration, which could be assigned to their higher content in the sulfur-containing compounds, such as 2-methyl-3-furanthiol, furfuryl mercaptan and so on. Lower cysteine level leads to less pronounced meaty taste, while higher levels of cysteine increased the sulfur odor, so sample A had the weakest meaty taste while sample E had a strong sulphur odor with a pungent odor, which also led to the significant decrease of meaty taste, umami, mouthfulness, continuity, and total acceptance. This is consistent with the quantitative results of GC–MS. On the other hand, the bitter taste intensity was the highest in sample A. Among all the samples, D and E presented stronger umami and salty taste score, while B and C presented stronger meaty, umami, and mouthfulness taste score. In contrast, D and E showed lower bitter taste intensities, which could be due to the decline in the bitter peptides and amino acid contents, caused by cross-linking at greater extents upon heating. In general, the total acceptance of samples B and C was better than others.

Therefore, it can be noted that 0.25–0.75% range of addition of cysteine increased the meaty, caramel, umami, mouthfulness, and salty notes, and caused a decrease in bitter taste score. The results are consistent with previous research that sulfur-containing MRPs provided a characteristic continuity and mouthfulness flavor that contributed to aroma and taste (Ueda et al. 1997).

### Relationship between browning intensity, free amino acid, molecular weight, flavor compounds and sensory characteristics

**Fig. 6:**
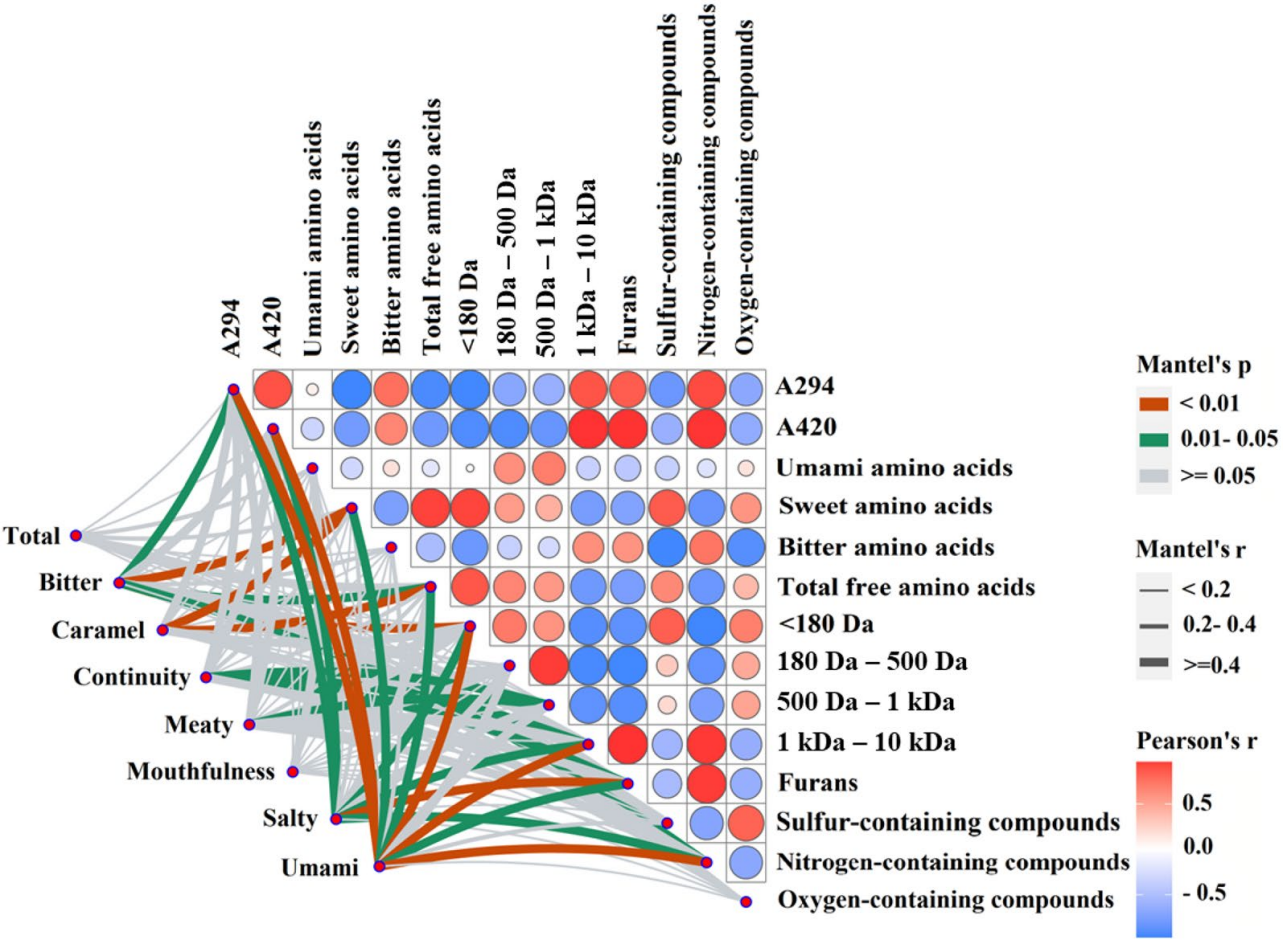
The correlations between sensory characteristics and physicochemical properties using Pearson and Mantel tests analysis

Mantel test was performed to identify correlations between organoleptic characteristics and physicochemical indicators including browning intensity, free amino acid, molecular weight, and flavor compounds (Fig. 6). Color gradients were used to represent pairwise comparisons of physicochemical indicators, with red circles denoting positive correlations and blue circles denoting negative correlations. The darker the color, the stronger the correlation. In addition, the color and width of the line, which linked the organoleptic characteristics and physicochemical indicators, represented statistical significance and Mantel’s r statistic, respectively (Dai et al. 2022).

There were significant (*p* < 0.05) positive correlations between the content of furan, nitrogen-containing compounds, and peptide with molecular weight above 1 kDa, and between bitter amino acids and nitrogen-containing compounds. Positive correlations were also found between browning intensity and peptide fraction ranging 1 kDa–10 kDa. However, the contents of furans and nitrogen-containing compounds negatively correlated with peptide fraction ranging 180–1,000 Da, and negative correlation was also found between umami amino acids and peptide fraction ranging 180 Da–1 kDa, and between bitter amino acids and sulfur-containing compounds.

Mantel test results showed that organoleptic characteristics had strong associations (Mantel’s *r* > 0.4, *p* < 0.01) with physicochemical index, especially nitrogen-containing compounds, furans, and peptide fraction ranging 500 Da–1 kDa. While weak correlation (Mantel’s *r* < 0.2, 0.01 < *p* < 0.05 or *p* > 0.05) was found between organoleptic characteristics and oxygen-containing. Interestingly, there were significant correlations between meaty flavor and peptides with peptide fraction ranging 500 Da–1 kDa, and similar correlation was found between bitter flavor and nitrogen-containing compounds. Additionally, total acceptance, mouthfulness, and continuity were significantly associated with umami amino acids. Overall, the organoleptic characteristics of MRPs are closely related to the physicochemical properties and cysteine has significant effects on the physicochemical and organoleptic characteristics of MRPs.

## Conclusions

The present study demonstrated that cysteine addition could significantly affect the MRPs derived from the xylose–glycine–FPHs Maillard reaction systems. The cysteine could significantly inhibit the browning and the cross-linking of free amino acids and reduce sugar contents, which also reduced the generation of bitter FAA contents, but improved sweet and umami FAA contents. MW distribution showed that higher concentration of cysteine suppressed the cross-linking of peptide fraction ranging 180 Da– kDa and accelerated the degradation of peptides with MW higher than 1 kDa. Meanwhile, the sensory evaluation indicated that cysteine could improve the organoleptic characteristics through increasing the meaty, umami, and sweetness, and reducing the bitterness of the MRPs. Volatile compounds analysis by GC–MS and GC–IMS confirmed that cysteine favored the formation of sulfur- and oxygen-containing compounds, but not conducive to the formation of furans and nitrogen-containing compounds. The sensory characteristics of MRPs are closely related to the physicochemical properties by Mantel test results. Overall, the physicochemical and organoleptic characteristics of MRPs are closely related to the concentration of cysteine and 0.25–0.75% range of addition of cysteine could significantly improve the physicochemical and organoleptic characteristics of MRPs. These results provide a better understanding of the key factors that would influence the physicochemical and organoleptic characteristics of MRPs and are beneficial for the production of FPHs flavor enhancers in the food industry.

## Data Availability

All data obtained and analyzed in this study are presented within this published article.

## Abbreviations

FPHs: Fish protein hydrolysates
MRPs: Maillard reaction products
FAAs: Free amino acids
FTIR: Fourier transform infrared
MW: Molecular weight
PCA: Principal component analysis
